# Hopfield neural network model for explaining double dissociation in semantic memory impairment

**DOI:** 10.1186/1471-2202-14-S1-P233

**Published:** 2013-07-08

**Authors:** Shin-ichi Asakawa, Ikuko Kyoya

**Affiliations:** 1 Tokyo Woman's Christian University, Zempukuji 2, 6, 1, Suginami, 1678585 Tokyo, Japan; 2 Ritsumeikan University, JitohinKitamachi, 56, 1, Kita, 6038577 Kyoto, Japan

## 

The purpose of this study was to look for possibility whether Hopfield model can be one of candidates of models for human semantic memory organization. In the field of cognitive psychology, the generalized context model, proposed by Nosofsky, has been considered as a promising one. This model insists that category judgment of human subjects depend upon all memory traces previous experienced. Being analogous Hopfield neural network model with the generalized context model, it is suggested that human semantic memory might be formulated using Hopfield model. It is well-known in neuropsychology that animate and inanimate concepts are separately declined by brain damages. Warrington and her colleagues proposed the "visual/functional" hypothesis, that the knowledge about visual and functional features are separately represented in our brains. Animate objects would share more visual properties than inanimate objects. On the other hand, inanimate objects are often defined by their functional features. According to this visual/functional hypothesis, Farah and McClelland (1991) proposed a neural network model to explain the double dissociation. They decided the number and ratio of processing units to deal with functional and visual properties from their psychological experiment. The results succeeded in showing category specificity, when they destroyed the network. However, it can be pointed out that their results was be obvious and no simulations were needed. The reason is that if animate objects/concepts had sent information to visual semantic memory more than those of inanimate objects, then animate specific category disorders must emerge inevitably when semantic memory would suffer damages. There are no needs for discussions nor simulations about this. However, no considerations about the ratio and validity had not been conducted so far. Tyler et al constructed a stimulus set and tried to explain the double dissociation to use this data set. In other words, Farah & McClelland sought the origin of double dissociation from intrinsic semantic memory organization. On the other hand, Tyler sought it from external stimulus structure. However, the reason and/or the degree of contribution of both factors have still remained unsolved. Therefore, mathematical considerations and simulation were conducted. The result showed that the ratio of neuron group, which dealt with, particular information might affect on the performance of the network. This parameter has higher contribution than the external data structure. If so, when brain damages would destroy this area, concept of animate objects might destroy in accordance with the degree of damages. However, we must remember that the validity of Farah and McClelland's ratio must be justified by all possible methodologies. In addition, we might not discard the Tyler structure as the origin of category specificity. It seems to be plausible that Tyler structure might be learned day by day, and then the ratio of Farah and McClelland as semantic memory organization might emerge as human semantic memory representation.

